# Diet quality, diet-related factors and disability status among male adults of reproductive age in the USA

**DOI:** 10.1017/S1368980023001222

**Published:** 2023-10

**Authors:** Andrea L Deierlein, Jaqueline Litvak, Chang Liu, Cheryl R Stein

**Affiliations:** 1 School of Global Public Health, New York University, New York, NY, USA; 2 Department of Child and Adolescent Psychiatry, New York University Grossman School of Medicine, New York, NY, USA

**Keywords:** Disability, Males, Healthy Eating Index, Nutrition

## Abstract

**Objective:**

To examine diet quality and diet-related factors among male adults of reproductive age with and without disabilities.

**Design:**

Cross-sectional data from the National Health and Nutrition Examination Surveys, 2013–2018.

**Setting:**

Disability was reported as serious difficulty hearing, seeing, concentrating, walking, dressing and/or running errands due to physical, mental or emotional conditions. Diet quality was assessed by the Healthy Eating Index (HEI)-2015 and diet-related factors included self-rated diet healthfulness, food security and food assistance programmes. Multivariable linear regression estimated differences in HEI-2015 scores. Multivariable Poisson regression estimated adjusted prevalence ratios (aPR) and 95 % CI for diet-related factors.

**Participants:**

In total, 3249 males, 18–44 years; of whom, 441 (13·4 %) reported having disabilities.

**Results:**

Compared with males without disabilities, those with disabilities had a 2·69-point (95 % CI: –4·18, –1·20) lower mean total HEI-2015 score and approximately one-third to half of a point lower HEI-2015 component scores for greens and beans, total protein foods, seafood and plant proteins, fatty acids and added sugars. Males with any disabilities were more likely to have low food security (aPR = 1·57; 95 % CI: 1·28, 2·92); household participation in food assistance programmes (aPR = 1·61; 95 % CI: 1·34, 1·93) and consume fast food meals during the previous week (1–3 meals: aPR = 1·11; 95 % CI: 1·01–1·21 and 4 or more meals: aPR = 1·18; 95 % CI: 1·01–1·38) compared with males with no disabilities.

**Conclusions:**

Factors affecting diet and other modifiable health behaviours among male adults of reproductive age with disabilities require further investigation. Health promotion strategies that are adaptive to diverse populations within the disability community are needed.

Diet plays an influential role for overall health and chronic disease development throughout the life course. Nutrient intakes during adolescence and early adulthood are particularly important for reproductive health outcomes^(1)^, mental health and cognitive function^(2)^ and cardiometabolic health risk factors^(3)^. Persons with disabilities often report poorer health status and have higher rates of chronic health conditions compared with their counterparts without disabilities^(4,5)^. There are numerous contributors to these health disparities, including socio-economic status, lifestyle behaviours, healthcare access and receipt of preventive healthcare services^(6–8)^. Additionally, an increased risk of household food insecurity is consistently associated with disability status^(9)^; however, there remains limited investigation of dietary intakes and other diet-related factors among persons with disabilities, especially young adults.

Findings from descriptive studies of nutrient intakes or nutritional behaviours among small convenience samples of adults with physical disabilities (e.g. spinal cord injury)^(10,11)^, adults with intellectual or developmental disabilities^(12,13)^ and Paralympic athletes^(14)^ suggest that many of these individuals do not meet dietary recommendations for some vitamins and minerals, while exceeding recommendations for other nutrients, like Na, fat and alcohol. The U.S. National Health and Nutrition Examination Surveys (NHANES) is a nationally representative program of studies that uniquely collects information on both disability status and dietary intakes^(15)^. Among adults (ages 20 years and older) in NHANES 2007–2010, nutrient intakes from food and dietary supplements were compared between those with and without disabilities and across five categories of disability (based on nineteen questions about difficulties with activities)^(16)^. Adults with disabilities, especially those with disabilities related to activities of daily living, were more likely to have dietary intakes that fell outside of national recommendations compared with those without disabilities. Differences between males and females or age categories were not examined^(16)^.

Beginning in 2013, NHANES added a disability questionnaire with six specific questions about difficulties related to hearing, seeing, concentrating, walking, dressing and/or running errands due to physical, mental or emotional conditions. In previous analyses, we examined diet quality and diet-related factors by disability status among female adults of reproductive age (18–44 years) in NHANES 2013–2018^(17)^. Despite few differences in diet quality scores between females with and without disabilities, those with any disabilities were more likely to rate their diet health as poor, have low food security, participate in food assistance programmes and consume frozen foods^(17)^. There has been little focus on the nutritional health of male adults of reproductive age or how it may differ from that of their female counterparts. For the current analyses, our objective was to address this gap by examining diet quality and diet-related factors among male adults of reproductive age in NHANES 2013–2018.

## Methods

### Study population

Data were from 3754 male adults aged 18–44 years who participated in NHANES cycles 2013–2014, 2015–2016 and 2017–2018. NHANES is conducted by the National Center for Health Statistics and collects socio-demographic, lifestyle, clinical, laboratory and nutrition information from individuals of all ages during interviews and medical exams^(15)^. For the current analyses, males were excluded if they had missing (*n* 286) or unreliable dietary data (*n* 15 flagged as unreliable by NHANES) or were missing information on covariates (*n* 25). There were 3249 males included in the final analytic sample. Informed consent was obtained from all participants, and approval for the studies was obtained from the National Center for Health Statistics Research Ethics Review Board. Descriptions of NHANES and data collection procedures are described elsewhere^(15)^. The current study was exempted from human subjects review by the New York University Institutional Review Board.

### Disability status

The disability questionnaire consisted of six questions regarding self-reported difficulty (yes or no) with activities due to physical, mental or emotional conditions: (1) ‘deaf or serious difficulty hearing’; (2) ‘blind or serious difficulty seeing even when wearing glasses’; (3) ‘serious difficulty concentrating, remembering, or making decisions’; (4) ‘serious difficulty walking or climbing stairs’; (5) ‘difficulty dressing or bathing’ and (6) ‘difficulty doing errands alone such as visiting a doctor’s office or shopping’. Self-reported disability status was dichotomised as none or any disabilities. To be consistent with previous NHANES analyses among female adults of reproductive age^(4,17)^, any disabilities were further categorised as four disability types: sensory (questions 1 and 2); cognitive (question 3); movement (question 4) and self-care (questions 5 and 6). Associations for males who reported any disabilities, only one type of disability and two or more types of disabilities were compared with males with no disabilities.

### Diet quality

The dietary interview component of NHANES, What We Eat in America, is conducted by the U.S. Department of Agriculture and U.S. Department of Health and Human Services^(15)^. It consists of two 24-h dietary recalls, during which participants report food and beverage consumption within the previous 24 h. The first 24-h dietary recall was collected during an in-person interview by trained staff who were fluent in Spanish and English. The second 24-h dietary recall was administered by telephone approximately 3–10 d after the first recall^(15)^. Approximately 18 % of males did not complete the second 24-h dietary recall (*n* 591); therefore, only food and beverage intakes (excluding vitamin and supplement use) collected from the first 24-h dietary recall were included.

Diet quality specific to the first 24-h dietary recall was assessed using The Healthy Eating Index (HEI)-2015. HEI-2015 is a measure of diet quality based on adherence to the 2015–2020 Dietary Guidelines for American^(18)^, which most reflects the U.S. dietary recommendations at the time of NHANES data collection. Better HEI scores are associated with reduced chronic disease risk and mortality in male adults and the general population^(19,20)^. HEI-2015 scores were calculated using the simple HEI scoring algorithm method with publicly available SAS code from the National Cancer Institute’s Epidemiology and Genomics Research Program^(21,22)^. For these analyses, all HEI scores should be interpreted as representing dietary intakes and diet quality on a given day.

The HEI-2015 has thirteen components, nine adequacy components and four moderation components that are summed for an overall score ranging from 0 to 100 points^(18)^. A higher total HEI-2015 score indicates better diet quality^(18)^. The nine adequacy components (total sxity points) are: total fruits (five points), whole fruits (five points), total vegetables (five points), greens and beans (five points), total protein foods (five points), seafood and plant proteins (five points), whole grains (ten points), dairy (ten points) and fatty acids (ratio of polyunsaturated fats and monounsaturated fats to saturated fat, ten points). With the exception of the fatty acids component, adequacy components were scored based on nutrient density per 1000 calories (e.g. cups of whole fruit per 1000 calories). For all of the adequacy components, a higher score indicates higher intakes of that component. The four moderation components (total forty points) are: refined grains (ten points), Na (ten points), added sugars (ten points) and saturated fats (ten points). The refined grains and Na components were scored based on amounts per 1000 calories; the saturated fats and added sugars components were scored based on percentage of total energy intakes. For all of the moderation components, a higher score indicates lower intakes of that component^(18)^.

### Diet-related factors

Several factors related to diet were examined. Participants rated the healthfulness of their overall diet as excellent, very good, good, fair or poor (Diet Behaviour and Nutrition questionnaire). Adult food security status was based on responses to the ten-item U.S. Food Security Survey Module (FFQ)^(23)^. Responses were categorised as full (no affirmative responses to any of the ten items), marginal (1–2 affirmative responses) or low/very low (three or more affirmative responses) food security^(23)^. Participation in governmental food assistance programmes was based on receipt of ‘Supplemental Nutrition Assistance Program (SNAP) or Food Stamp benefits’ within the previous year (self or someone in the household, yes or no) (Food Security questionnaire)^(24)^. SNAP is a federal program that provides monthly benefits for low-income households to purchase food^(24)^. Participants responded to questions regarding their frequency of consumption of different types of meals or foods (Diet Behaviour and Nutrition questionnaire): total number of meals (breakfast, lunch or dinner) prepared away from home during the previous 7 d (e.g. restaurants, fast food places, food stands, grocery stores, vending machines; categorised as none, 1–3 meals and 4 or more meals); number of meals prepared away from home from a fast food or pizza place during the previous 7 d (none, 1–3 meals and 4 or more meals); number of times that ready-to-eat foods from grocery stores were consumed in the past 30 d (e.g. salads, soups, chicken and sandwiches; categorised as none, 1–4 times and five or more times) and number of times that frozen meals or frozen pizzas were consumed in the past 30 d (none, 1–four times and five or more times). In NHANES 2017–2018 questions were added (Diet Behaviour and Nutrition questionnaire) regarding participants’ status as the main meal planner/preparer for their household (yes or no) or if they shared in the planning or preparing of meals with someone else (yes or no), and participants’ status as the main food shopper (yes or no) or if they shared in food shopping with someone else (yes or no).

### Socio-demographic characteristics

Age (years), race and ethnicity (categorised in NHANES as non-Hispanic white, non-Hispanic Black, Mexican American, Other Hispanic and Other Race including multi-racial), educational status (< high school graduate, high school graduate/some college and college graduate), ratio of family income to poverty (expressed as a percentage, <= 100 %, 101–200 %, 201–300 % and > 300 %), marital status (single, married/living with partner) and cigarette smoking (never, former and current) were self-reported. During an in-person medical exam, participants’ weight (kilograms, kg) and height (meters, m) were measured by trained health technicians using standardised procedures while participants wore examination gowns and no shoes^(15)^. BMI (BMI, kg/m^2^) was calculated using these measurements.

### Statistical analysis

Unweighted counts, means with standard errors (se, continuous variables) and proportions (categorical variables) were calculated. Bivariate associations of disability status (no disabilities, any self-reported disabilities, only one self-reported disability, two or more self-reported disabilities) and participant characteristics, total HEI-2015 scores and diet-related factors were tested using *χ* ^2^ tests (categorical variables) and *t* tests (continuous variables). Multivariable linear regression estimated associations of disability status and continuous HEI-2015 total and component scores. Multivariable Poisson regression estimated associations (prevalence ratios, PR, and 95 % CI) of disability status and categorical diet-related factors. The reference for all models was male adults with no disabilities. Models were adjusted for age, race and ethnicity, education, marital status, BMI and smoking status based on previous literature^(16,17)^ and directed acyclic graph analysis^(25)^. Ratio of family income to poverty was missing for 301 (9 %) males. Multivariable regression models were examined with and without adjustment for this variable. There were no appreciable differences in the magnitude or precision of effect estimates; therefore, final models did not include this variable. Statistical significance was set at *P* < 0·05. All analyses were statistically weighted as required for the complex survey design of NHANES using Stata version 15.1^(26)^.

## Results

Of 3249 male adults of reproductive age, 441 (13·4 %, weighted) reported having any disability: 290 (8·3 %) reported having only one type of disability and 151 (5·1 %) reported having two or more types of disabilities. Some differences were observed in the distributions of socio-demographic characteristics by disability status. Males with any disabilities were less likely to be college graduates, from households with higher incomes and married or living with a partner; they were more likely to be current smokers (Table 1).

**Table 1:** Distributions of socio-demographic characteristics and health behaviours among adult males of reproductive age (18–44 years) with and without self-reported disabilities, national health and nutrition examination surveys (NHANES), 2013–2018 (*n* 3249)

Characteristic	No disabilities		Any disabilities		*P* †	One disability		*P* ^2^	Two or more disabilities		*P* †	*P* ‡
	*n*	%*	*n*	%*		*n*	%*		*n*	%*		
Total	2808	86·6	441	13		290	8		151	5		
	Mean	se	Mean	se		Mean	se		Mean	se		
Age (years)	30·3	0·20	30·7	0·46	0·47	30·3	0·56	0·97	31·2	0·82	0·29	0·40
18–25	943	33	136	28		106	34		30	20		
26–35	1024	38	163	41	0·25	98	36	0·82	65	50	0·02	0·05
36–44	841	29	142	30		86	30		56	30		
	*n*	%	*n*	%		*n*	%		*n*	%		
Race and Ethnicity					0·79			0·68			0·39	0·25
Non-Hispanic White	929	56	190	14		111	54		79	64		
Non-Hispanic Black	583	12	83	8		61	14		22	9		
Mexican American	463	13	77	57		54	15		23	12		
Other Hispanic	262	8	40	12		29	9		11	5		
Other Race, including multi-racial	571	12	51	10		35	9		16	10		
Education					< 0·0001			< 0·0001			< 0·0001	0·01
< High school	527	13	131	22		91	25		40	18		
High school graduate	1594	58	276	68		172	63		104	78		
College graduate	687	29	34	10		27	13		7	5		
Ratio of family income to poverty level (%)§					0·0001			0·002			0·02	0·42
<= 100 %	543	15	126	23		79	22		47	24		
101–200 %	649	22	128	30		82	31		46	28		
201–300 %	450	18	55	16		36	13		19	21		
> 300 %	900	46	97	31		68	34		29	26		
Marital status					0·0002			0·007			0·005	0·43
Single	1327	47	262	61		174	59		88	65		
Married/Living with partner	1481	53	179	39		116	41		63	35		
	Mean	se	Mean	se		Mean	se		Mean	se		
BMI	28·6	0·22	29·3	0·48	0·17	29·6	0·51	0·07	28·8	0·86	0·86	0·41
< 18·5	53	2	11	2		NR||			NR||			
18·5–< 25	907	30	119	28	0·77	78	25	0·25	41	34	0·79	0·32
25–< 30	910	32	131	31		87	30		44	33		
>= 30	938	36	180	39		116	43		64	32		
	*n*	%	*n*	%		*n*	%		*n*	%		
Smoking status					< 0·0001			< 0·0001			< 0·0001	0·53
Current	435	14	126	31		85	30		41	33		
Former	647	27	111	27		70	25		41	29		
Never	1726	59	204	42		135	45		69	38		

se, standard error; NR, not reported.

* Percentages were adjusted for NHANES survey weights.

† Reference is males with no disabilities.

‡ Reference is males with only one type of disability.

§ *n* 301 (9 %) males missing information on household income.

|| Cell sizes <= 10.

Among male adults of reproductive age without disabilities, the mean unadjusted total HEI-2015 score was 48·16 (se = 0·38) compared with mean unadjusted scores of 43·68 (se = 0·73), 44·12 (se = 0·82) and 42·97 (se = 1·43) among males with any disabilities, only one type of disability and two or more types of disabilities, respectively (Table 2). These observed differences remained after adjusting for covariates. The adjusted mean total HEI-2015 scores among males with any disabilities, only one type of disability and two or more types of disabilities were lower than the score for males without disabilities by 2·69 points (95 % CI: –4·18, –1·20), 2·28 points (95 % CI: –3·87, –0·68) and 3·37 points (–6·14, –0·60), respectively. Compared with males with no disabilities, those with any disabilities had lower adjusted mean scores for greens and beans, total protein foods, seafood and plant proteins, fatty acids and added sugars (Table 2). In models stratified by number of types of disabilities, differences in adjusted mean HEI component scores tended to be greater (indicative of lower diet quality) for males with two or more types of disabilities than males with only one type of disability, when compared with males with no disabilities. Males with two or more disabilities had lower scores for total vegetables, greens and beans, total protein foods, seafood and plant proteins and added sugars, compared with males with no disabilities (Table 2).

**Table 2:** Unadjusted means and adjusted mean differences in total and component Health Eating Index (HEI)-2015 scores for a given day by disability status, categorised as no disabilities, any disability, only one disability, and two or more disabilities, among male adults of reproductive age (18–44 years) in the National Health and Nutrition Examination Surveys (NHANES), 2013–2018

	No disabilities (*n* 2808)			Any disabilities (*n* 441)				One disability (*n* 290)				Two or more disabilities (*n* 151)			
	Score range	Mean*	se	Mean*	se	*ß* †	95 % CI	Mean*	se	*ß* †	95 % CI	Mean*	se	*ß* †	95 % CI
Total HEI-2015 Score	0–100	48·16	0·38	43·68	0·73	−2·69	−4·18, –1·20	44·12	0·82	−2·28	−3·87, –0·68	42·97	1·43	−3·37	−6·14, –0·60
Total fruits	0–5	1·64	0·05	1·23	0·11	−0·21	−0·44, 0·02	1·26	0·13	−0·21	−0·46, 0·04	1·19	0·20	−0·21	−0·63, 0·20
Whole fruits	0–5	1·66	0·06	1·17	0·12	−0·24	−0·51, 0·02	1·17	0·14	−0·27	−0·55, 0·004	1·18	0·23	−0·20	−0·68, 0·27
Total vegetables	0–5	2·72	0·05	2·34	0·11	−0·22	−0·46, 0·03	2·54	0·12	−0·03	−0·29, 0·24	2·01	0·16	−0·54	−0·87, –0·20
Greens and beans	0–5	1·50	0·06	0·91	0·11	−0·32	−0·58, –0·06	1·08	0·14	−0·19	−0·49, 0·11	0·64	0·16	−0·53	−0·88, –0·18
Total protein foods	0–5	4·33	0·03	4·00	0·11	−0·28	−0·51, –0·05	4·03	0·14	−0·26	−0·54, 0·03	3·95	0·19	−0·32	−0·70, 0·06
Seafood and plant proteins	0–5	2·19	0·07	1·59	0·15	−0·39	−0·70, –0·09	1·65	0·16	−0·33	−0·65, –0·02	1·48	0·26	−0·50	−1·00, –0·02
Whole grains	0–10	2·01	0·09	1·73	0·16	−0·01	−0·35, 0·34	1·62	0·18	−0·12	−0·52, 0·29	1·90	0·36	0·18	−0·51, 0·87
Dairy	0–10	5·05	0·10	4·83	0·18	−0·07	−0·46, 0·32	4·78	0·20	−0·12	−0·59, 0·35	4·90	0·40	0·01	−0·69, 0·71
Fatty acids	0–10	4·76	0·09	4·21	0·20	−0·44	−0·88, –0·01	4·34	0·24	−0·31	−0·77, 0·15	4·01	0·40	−0·67	−1·52, 0·19
Refined grains	0–10	5·80	0·11	5·78	0·23	−0·20	−0·71, 0·32	5·48	0·27	−0·43	−0·97, 0·12	6·27	0·44	0·18	−0·81, 1·17
Na	0–10	4·03	0·12	4·39	0·25	0·09	−0·47, 0·65	4·31	0·29	0·04	−0·60, 0·68	4·52	0·46	0·18	−0·76, 1·12
Added sugars	0–10	6·77	0·10	5·68	0·26	−0·50	−0·98, –0·02	6·02	0·33	−0·22	−0·89, 0·45	5·15	0·42	−0·96	−1·76, –0·15
Saturated fats	0–10	5·69	0·10	5·83	0·21	0·10	−0·39, 0·60	5·85	0·24	0·16	−0·36, 0·68	5·79	0·41	0·01	−0·89, 0·91

se, standard error; CI, confidence interval.

* Unadjusted mean.

† Mean difference in HEI-2015 scores from linear regression models adjusted for age, race and ethnicity, education, marital status, BMI and smoking status, reference group for all models is males with no disabilities.

Differences in distributions of diet-related factors were also observed by disability status (Table 3). Greater proportions of males with any disabilities were more likely report poor or fair self-rated diet health; very low or low food security status; household participation in food assistance programmes during the previous year; consume at least one fast food meal during the previous week and consume frozen foods or pizza at least once during the previous month. After adjustment for covariates (Table 4), some of these observed differences in diet-related factors by disability status remained. Males with any disabilities were more likely to have very low/low food security status (aPR, 1·57; 95 % CI: 1·28, 2·92); household participation in food assistance programmes (aPR, 1·61; 95 % CI: 1·34, 1·93) and consume at least one meal from fast food/pizza places during the past 7 d (1–3 meals: aPR, 1·11; 95 % CI: 1·01, 1·21 and 4 or more meals: aPR, 1·18; 95 % CI: 1·01, 1·38) compared with males with no disabilities. In models that stratified by number of types of disabilities, associations were similar for males with one disability and males with two or more disabilities compared with those with no disabilities. Diet-related factors of self-rated diet health; consumption of meals prepared away from home, ready-to-eat foods, and frozen meals/pizza and status as main food shopper and main meal planner/preparer were not associated with disability status.

**Table 3:** Distributions of diet-related factors among male adults of reproductive age (18–44 years) with and without self-reported disabilities, National Health and Nutrition Examination Surveys (NHANES), 2013–2018

Diet-related Factor	No disabilities		Any disabilities		*P* †	One disability		*P* †	Two or more disabilities		*P* †	*P* ‡
	*n*	%*	*n*	%*		*n*	%*		*n*	%*		
Self-rated diet health					0·003			0·06			0·001	0·10
Excellent/Very good	674	24	70	18		41	16		29	22		
Good	1137	41	144	33		103	39		41	24		
Fair	801	28	170	37		110	34		60	43		
Poor	196	7	57	11		36	12		21	11		
Food security status					< 0·0001			0·0001			< 0·0001	0·37
Full	1709	70	193	53		135	56		58	49		
Marginal	403	12	62	10		42	11		20	9		
Low/Very low	604	18	172	37		104	33		68	42		
Household food assistance (past year)					< 0·0001			< 0·0001			< 0·0001	0·40
No	2171	84	267	65		181	67		86	62		
Yes	637	16	174	35		109	33		65	38		
Number of meals prepared away from home (past 7 d)					0·20			0·07			0·12	0·03
None	330	10	67	12		37	11		30	14		
1–3	1090	38	181	40		125	46		56	31		
4 or more	1376	53	192	48		128	43		64	55		
Number of meals from fast food/pizza places (past 7 d)					0·03			0·03			0·01	0·003
None	475	20	38	12		20	10		18	14		
1–3	1286	53	215	54		155	63		60	39		
4 or more	711	28	121	35		78	27		43	47		
Number of times consumed ready-to-eat foods (past 30 d)					0·56			0·34			0·87	0·61
None	1749	61	267	60		173	61		94	58		
1–4	617	23	97	21		61	19		36	25		
5 or more	434	17	71	19		53	20		18	18		
Number of times consumed frozen meals/frozen pizza (past 30 d)					0·02			0·03			0·08	0·35
None	1644	56	224	47		150	50		74	43		
1–4	697	28	112	28		65	25		47	33		
5 or more	458	16	104	25		74	25		30	24		
Main meal planner/preparer§					0·32			0·66			0·10	0·12
No	523	64	98	59		63	67		35	48		
Yes	271	36	45	41		22	33		23	52		
Shares in meal planning/preparation§					0·15			0·22			0·75	0·59
No	331	40	62	48		36	52		26	43		
Yes	463	60	81	52		49	48		32	57		
Main food shopper§					0·58			0·85			0·49	0·54
No	520	66	94	62		58	65		36	58		
Yes	274	34	49	38		27	35		22	42		
Shares in food shopping§					0·10			0·18			0·97	0·37
No	313	36	62	45		37	52		25	35		
Yes	481	64	81	55		48	49		33	65		

* Percentages were adjusted for NHANES survey weights.

† Reference is males with no disabilities.

‡ Reference is males with only one disability.

§ Only collected in NHANES 2017–2018.

**Table 4:** Unadjusted and adjusted* Poisson regression models estimating associations of selected diet-related factors and disability status, categorised as no disabilities,† any disability, only one disability and two or more disabilities, among male adults of reproductive age (18–44 years) in the National Health and Nutrition Examination Surveys (NHANES), 2013–2018

Diet-related factor	Any disabilities				One disability				Two or more disabilities			
	PR	95 % CI	aPR	95 % CI	PR	95 % CI	aPR	95 % CI	PR	95 % CI	aPR	95 % CI
Self-reported diet health												
Excellent/Very good	REF		REF		REF		REF		REF		REF	
Fair	1·25	1·07, 1·47	1·13	0·95, 1·35	1·27	1·05, 1·54	1·11	0·91, 1·35	1·23	1·02, 1·50	1·17	0·94, 1·45
Poor	1·72	1·13, 2·60	1·04	0·73, 1·47	1·90	1·17, 3·06	1·07	0·73, 1·57	1·47	0·82, 2·65	0·99	0·56, 1·75
Food security status												
Full	REF		REF		REF		REF		REF		REF	
Marginal	1·11	0·78, 1·59	0·92	0·65, 1·28	1·14	0·72, 1·80	0·90	0·59, 1·37	1·06	0·64, 1·75	0·95	0·57, 1·59
Low/Very low	2·05	1·63, 2·58	1·57	1·28, 1·92	1·88	1·40, 2·53	1·41	1·10, 1·81	2·32	1·73, 3·11	1·84	1·39, 2·44
Household food assistance (past year)												
No	REF		REF		REF		REF		REF		REF	
Yes	2·14	1·73, 2·64	1·61	1·34, 1·93	2·01	1·59, 2·56	1·49	1·19, 1·85	2·34	1·72, 3·18	1·82	1·35, 2·47
Number of meals prepared away from home (past 7 d)												
None	REF		REF		REF		REF		REF		REF	
1–3	0·95	0·87, 1·05	0·98	0·89, 1·08	1·01	0·92, 1·10	1·04	0·94, 1·14	0·85	0·69, 1·03	0·86	0·70, 1·06
4 or more	0·94	0·87, 1·01	0·96	0·89, 1·04	0·94	0·85, 1·04	0·96	0·87, 1·06	0·93	0·84, 1·04	0·97	0·85, 1·10
Number of meals from fast food/pizza places (past 7 d)												
None	REF		REF		REF		REF		REF		REF	
1–3	1·13	1·02, 1·24	1·11	1·01, 1·21	1·18	1·07, 1·31	1·16	1·06, 1·28	1·00	0·83, 1·22	0·99	0·82, 1·20
4 or more	1·28	1·11, 1·49	1·18	1·01, 1·38	1·26	1·05, 1·50	1·14	0·96, 1·35	1·31	1·08, 1·58	1·23	0·99, 1·52
Number of times consumed ready-to-eat foods (past 30 d)												
None	REF		REF		REF		REF		REF		REF	
1–4	0·96	0·73, 1·26	0·98	0·74, 1·29	0·87	0·62, 1·22	0·89	0·63, 1·24	1·09	0·73, 1·63	1·12	0·74, 1·69
5 or more	1·14	0·83, 1·56	1·11	0·81, 1·53	1·15	0·84, 1·58	1·15	0·84, 1·57	1·10	0·63, 1·92	1·05	0·60, 1·83
Number of times consumed frozen meals/frozen pizza (past 30 d)												
None	REF		REF		REF		REF		REF		REF	
1–4	1·13	0·91, 1·40	1·09	0·88, 1·35	1·01	0·77, 1·31	0·99	0·77, 1·29	1·32	0·96, 1·83	1·24	0·88, 1·74
5 or more	1·51	1·14, 1·99	1·30	0·98, 1·72	1·46	1·10, 1·95	1·35	1·00, 1·81	1·59	1·10, 2·32	1·23	0·85, 1·76
Main meal planner/preparer‡												
No	REF		REF		REF		REF		REF		REF	
Yes	1·15	0·87, 1·52	1·10	0·81, 1·48	0·92	0·63, 1·35	0·87	0·61, 1·23	1·44	0·98, 2·12	1·41	0·95, 2·09
Shares in meal planning/preparation‡												
No	REF		REF		REF		REF		REF		REF	
Yes	0·87	0·70, 1·08	0·91	0·76, 1·09	0·80	0·53, 1·21	0·85	0·62, 1·16	0·95	0·66, 1·37	0·99	0·67, 1·46
Main food shopper‡												
No	REF		REF		REF		REF		REF		REF	
Yes	1·12	0·75, 1·66	1·04	0·69, 1·56	1·03	0·71, 1·51	0·96	0·65, 1·43	1·22	0·69, 2·17	1·14	0·61, 2·14
Shares in food shopping‡												
No	REF		REF		REF		REF		REF		REF	
Yes	0·86	0·71, 1·05	0·91	0·78, 1·06	0·75	0·45, 1·26	0·80	0·51, 1·26	1·00	0·80, 1·27	1·06	0·85, 1·32

PR, prevalence ratio; aPR, adjusted prevalence ratio; REF, reference.

* Models adjusted for age, race and ethnicity, education, marital status, smoking status and BMI.

† Reference for all models is males with no disabilities.

‡ Collected only during NHANES 2017–2018.

## Discussion

Among U.S. male adults aged 18–44 years, overall diet quality scores were poor regardless of disability status. Compared with males without disabilities, those with any disabilities had a nearly three-point lower mean total HEI-2015 score and approximately one-third to half of a point lower HEI-2015 component scores for greens and beans, total protein foods, seafood and plant proteins, fatty acids and added sugars. Males with any disabilities were more likely to have very low/low food security status, household participation in food assistance programmes and consume at least one weekly meal from a fast food/pizza place compared with males without disabilities.

Few studies have assessed dietary intakes among adults with disabilities. Inadequate intakes of select food groups (e.g. fruits and vegetables) or micronutrients have been reported in descriptive studies of adults with impaired vision^(27)^, paraplegia^(28)^ and cerebral palsy^(12)^. In analyses of U.S. national survey data, adults with any disabilities reported less frequent fruit and vegetable consumption during the previous month compared with those without disabilities (*P* < 0·05)^(29)^ and did not meet U.S. dietary guidelines for saturated fat, cholesterol and Na (excess consumption) or for fibre, vitamins A, C and D, Ca and potassium (insufficient consumption)^(16)^. Smaller studies among male disabled adult populations show comparable nutritional disparities and are consistent with findings in the current study. Male adults with lower limb amputations in the United Kingdom (mean age = 46 years, *n* 46) had higher intakes of sugars, total and saturated fat and Na compared with national health recommendations (*P* < 0·05 for all comparisons)^(30)^. In the U.S. (Chicago, Illinois), dietary intakes were assessed in healthy, community-dwelling male adults with chronic spinal cord injury (ages 20–59 years, *n* 95)^(11)^. Only one-third of the participants had intakes within national dietary recommendations for saturated fat, Na and fruits and vegetable intakes, while 18 %, 16 % and 12 % met recommendations for total fat, dairy products and fibre, respectively^(11)^. Additionally, they had significantly lower scores for HEI components (earlier version of HEI, not specified by authors) related to grains, fruit, dairy, total fat and having a varied diet (*P* < 0·05 for all comparisons) when compared with scores of male adults in NHANES 1999–2000^(11)^. Though direct comparisons of findings from previous studies and the current study cannot be made, collectively, they suggest that malnutrition is a concern among adults with disabilities. In the current study, low diet quality scores among males with disabilities may be at least partially attributed to greater prevalence of food insecurity, participation in government food assistance programmes and consumption of fast foods, all of which have been associated with lower diet quality^(31–33)^.

There has been little inquiry regarding whether nutritional health (encompassing food security, physical and economic access to food, dietary behaviours and dietary intakes) varies between males and females with disabilities, especially younger adult populations. Similar to the current findings for male adults of reproductive age with disabilities, their female counterparts (in NHANES 2013–2018) were also more likely to report low household food security and participation in food assistance programmes; however, diet quality did not markedly vary by disability status among females^(17)^. Differences for several other diet-related factors were observed. Females with disabilities were more likely to perceive poor dietary health, more likely to consume frozen foods and less likely to be the main meal planners/preparers or main food shoppers for their households^(17)^; males with disabilities were more likely to consume fast food meals on a weekly basis. The contrasting associations for diet quality and diet-related factors between female and male adults of reproductive age with disabilities (compared with their respective counterparts without disabilities) suggest they have different needs that should be identified and addressed within nutrition interventions. These needs may be related to social determinants of health, as well as eating and lifestyle behaviours, attitudes and beliefs or nutrition knowledge. Studies have explored how psychological, social, physical and environmental variables influence nutritional and other health behaviours among females with disabilities,^(34–36)^ and subsequent health promotion strategies have proven successful^(36,37)^. For example, a disability- and gender-responsive weight management pilot intervention for women with mobility impairments improved their diet, physical activity and self-efficacy for diet and physical activity, as well as reduced body weight and waist circumference^(37)^. Continued research is necessary to understand barriers and facilitators of achieving healthy dietary intakes (and other health behaviours) and to develop health promotion strategies that are adaptive to diverse populations within the disability community.

Diet is a modifiable health behaviour. Greater attention should be paid to helping individuals with disabilities meet nutritional recommendations, beginning early in life and persisting throughout the life course. Disparities in overweight and obesity by disability status are apparent during childhood and continue into adulthood^(38,39)^. Adolescents and adults of reproductive age with disabilities are more likely to have chronic health conditions, including type 2 diabetes, hypertension and depression, compared with those without disabilities^(4,5,39,40)^. All of these health conditions may be ameliorated or prevented with dietary modifications^(41)^. Dietary screening and counseling may be overlooked during healthcare visits due to prioritisation of other healthcare needs or lack of healthcare provider knowledge on these topics. Several strategies have been proposed to address health promotion and wellness among persons with disabilities within the healthcare system, which should emphasise healthy eating and food access and availability^(42)^. These strategies include providing educational and practical training for primary care physicians and rehabilitation medicine specialists to support patients in wellness activities^(43,44)^; incorporating registered dietitian nutritionists into healthcare teams to provide individualised counseling for patients and their caregivers^(17)^ and establishing hospital-led, community-based programmes to transition patients from hospital care to independently managing their health and well-being^(45)^. In-person and telehealth interventions for self-esteem, physical activity and weight management have also been developed in various populations with disabilities^(46,47)^ and should be tested for their effectiveness in improving dietary intakes.

The major strength of this study is the use of U.S. survey data that collects information on disability status, detailed dietary intakes and other variables related to diet and health behaviours. Limitations include the cross-sectional study design and most of the study variables were self-reported. Questions about disability status only asked about whether or not there were serious difficulties with functional domains; they did not establish specific cause or severity of disability. Due to limited sample sizes, associations could not be examined by type of disability or other socio-demographic factors. Caution should be used when interpreting the HEI-2015 scores. These scores were calculated from a single 24-h dietary recall and are subject to biases related to self-reported data, including errors related to misreporting^(48)^. HEI-2015 scores based on 24-h recall data are similar to those from observed intakes^(49)^; however, these scores represent diet quality for a specific day rather than usual or habitual diet quality over time (e.g. variability by days, months and seasons)^(22,48)^. Lastly, the simple scoring algorithm method used to generate HEI-2015 scores does not account for measurement error, episodic food intakes, skewness or the correlation between dietary constituents and energy intakes^(22)^.

### Conclusions

Male adults of reproductive age with disabilities had lower diet quality and were more likely to report low food security, household use of food assistance programmes and weekly consumption of fast foods compared with those without disabilities. This is of concern because health behaviours and general health status during adolescence and early adulthood greatly influence fecundity and reproductive outcomes, personal short- and long-term physical and mental health, as well as the health and development of future generations. Factors affecting dietary intakes and related behaviours among male adolescents and adults of reproductive age with disabilities require further investigation. Health promotion and wellness programmes that include flexible strategies for overcoming barriers to healthy eating are necessary for reducing the existing health disparities faced by all individuals with disabilities.
